# Paleobotany reframes the fiery debate on Australia's rainforest edges

**DOI:** 10.1111/nph.20301

**Published:** 2024-11-27

**Authors:** Peter Wilf, Robert M. Kooyman

**Affiliations:** ^1^ Department of Geosciences and Earth and Environmental Systems Institute Pennsylvania State University University Park PA 16802 USA; ^2^ IUCN/SSC Global Tree Specialist Group Botanic Gardens Conservation International Richmond TW9 3BW UK; ^3^ Department of Biological Sciences Macquarie University Sydney NSW 2109 Australia; ^4^ Research Centre for Ecosystem Resilience Royal Botanic Gardens and Domain Trust Sydney NSW 2000 Australia

**Keywords:** *Eucalyptus*, fire, Laguna del Hunco, paleobotany, rainforest margins, rainforest restoration, Wet Tropics, World Heritage

## Abstract

The tall eucalypt forests (TEFs) of the Australian tropics are often portrayed as threatened by ‘invasive’ neighboring rainforests, requiring ‘protective’ burning. This framing overlooks that Australian rainforests have suffered twice the historical losses of TEFs and ignores the ecological and paleobiological significance of rainforest margins. Early Eocene fossils from Argentina show that biodiverse rainforests with abundant *Eucalyptus* existed > 50 million years ago (Ma) in West Gondwana, shaped by nonfire disturbance factors such as landslides and volcanic flows. Humid volcanic environments with eucalypts were also present in eastern Australia over much of the Cenozoic. The dominance of fire‐adapted eucalypts appears to be geologically recent and is linked to Neogene C_4_ grassland expansion, Pleistocene climate cycles, and human activity. We suggest that characterizing TEFs and rainforests as adversarial results from misinterpreting the evolutionary history and expansion‐contraction dynamics of a single humid forest system, whose features are now heavily modified by human activities. The resulting management practices damage the outstanding World Heritage values and carbon storage of affected areas and thus have impacts far beyond Australia. The fossil evidence shows that rainforest margins preserve ancient, still evolving, and globally significant forest interactions that should be prioritized for restoration and research.

## Introduction: the problem of rainforest edges

**Fig. 1:**
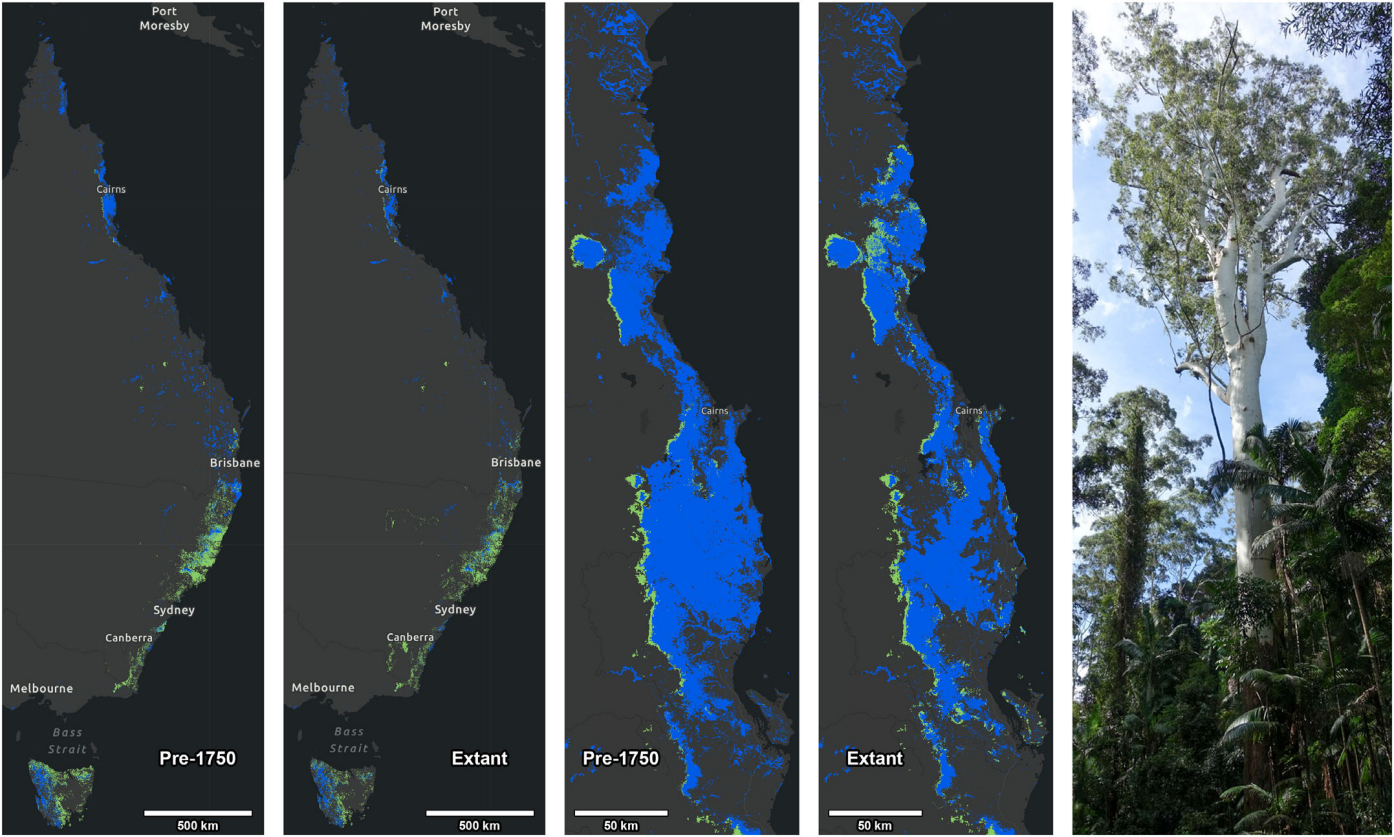
Estimated pre‐1750 vs extant cover for rainforests and vine thickets (blue) and tall eucalypt forests (TEFs, green) for eastern Australia, with detailed views of the Wet Tropics region. Modern city locations are shown pre‐1750 for positional reference. At right, an emergent Rose Gum (*Eucalyptus grandis*) in rainforest along Rummery Road, Nightcap National Park. Cover maps built in ArcGIS Pro 3.3 using National Vegetation Information System (NVIS 6.0) Major Vegetation Groups (MVGs) 1 and 2 defined at https://www.dcceew.gov.au/environment/land/publications/nvis‐fact‐sheet‐series‐4‐2; the NVIS pre‐1750 estimates and extant data are available at https://www.dcceew.gov.au/environment/land/native‐vegetation/national‐vegetation‐information‐system. The NVIS data indicate losses since 1750 of 28% of Australian rainforests and vine thickets (MVG1) and 18% of TEFs (MVG2), similar to the findings of Bradshaw (2012: fig. 3). Areas of TEFs in central Victoria are missing because they were classified (incorrectly) in MVG3 by NVIS, with negligible influence on the overall loss estimates because they were absent from both the historical and extant datasets.

Once distributed over most of the continent (Hill, 1994), Australia's remaining rainforests now cover only *c*. 0.5% of the land area, fragmented into a ‘mesic archipelago’ in the eastern portion of the country (Fig. 1; Kanowski *et al*., 2009; Byrne *et al*., 2011; Kooyman *et al*., 2013). Frequently adjoining and intermixing with the rainforest are wet, tall eucalypt forests (TEFs, often known in Australia as ‘wet sclerophyll forests’; > 30 m in height). The TEFs are dominated by trees in *Eucalyptus* s.l., collectively known as ‘eucalypts’ (*Eucalyptus*, *Angophora* and *Corymbia*), and other Myrtaceae, and they occur in humid climates similar to rainforests. They occupy *c*. 0.75% of Australia, including a version of the TEF in Western Australia without neighboring rainforest not discussed further here (e.g. Wardell‐Johnson *et al*., 2017). The rainforest–TEF interface is mediated by fire, climate, topography, edaphic factors, and plant traits (Beadle & Costin, 1952; Ashton, 1981; Ash, 1988; Wardell‐Johnson *et al*., 1997; Benwell, 2024; Fensham *et al*., 2024). The transition is sharp in the tropics, comprising a thin, well‐marked TEF fringe along the western rainforest edge. It becomes more diffuse to the southeast, where TEFs are concentrated closer to the coast, and rainforests are patchy; further south, rainforests are concentrated in the humid climate zone of western Tasmania (Fig. 1).

Despite their small areal extent, Australian rainforests are well‐recognized globally for their biological riches, including high faunal and floral endemism, incidence of threatened species, and extraordinary representation of ancient lineages known from fossils with biogeographic connections to ancient Gondwana, the rest of Australasia, and maritime Southeast Asia (Nix & Switzer, 1991; Kooyman *et al*., 2014; WTMA, 2014, 2016). The TEFs also support significant biodiversity (WTMA, 2014, 2016). These universal values supported the World Heritage inscription of rainforests and adjoining TEFs in areas including the Gondwana Rainforests of Australia and the Wet Tropics of Queensland; the latter ranked sixth in ‘irreplaceability’ among over 170 000 protected areas world‐wide because of its biodiversity and high concentration of threatened species (Le Saout *et al*., 2013; WTMA, 2014). Thus, eastern Australia's surviving humid forests have well‐established global importance and are not merely of regional interest.

Although numerous efforts are in place to protect them, Australian rainforests are far from ‘saved’. Rainforest historical clearing (28–35%) was approximately twice that of the TEFs (15–18%; Fig. 1; Bradshaw, 2012). Remote sensing analyses indicated 1.3% and 2.4% net losses for the 2000–2012 interval alone in Australia's tropical and subtropical rainforests, respectively (Hansen *et al*., 2013). The 2019 bushfires damaged large rainforest areas throughout eastern Australia, including the Gondwana Rainforests (Godfree *et al*., 2021; Fensham *et al*., 2024). Australian tropical rainforest plot analyses have shown doubled tree mortality risk over 35 yr, linked to increased temperatures and water deficits (Bauman *et al*., 2022).

Notwithstanding the perilous condition of Australian rainforests, a persistent narrative portrays their regrowth into TEFs as ‘invasions’ or ‘encroachments’ requiring containment by fire (Harrington & Sanderson, 1994; Harrington *et al*., 2000; Russell‐Smith *et al*., 2004; Stanton *et al*., 2014). This view is especially prevalent in the Wet Tropics, our focal region here (Fig. 1). The debate is not merely academic because it continues to dominate fire‐management policies (e.g. QPWS, 2012; Internet search strings such as ‘rainforest encroachment Australia’ recover numerous additional examples). For instance, Harrington & Sanderson (1994) stated that burning was necessary to preserve tropical TEFs and that ‘leaving it to nature is not an option’. Bradford (2018) recommended burning in Rose Gum (*E. grandis*; Fig. 1) forests of tropical Queensland every 3–5 yr, ‘regardless of the fire regimes that shaped these communities’. Australian rainforest ‘expansion’ (Fig. 1) has been characterized as an indicator of potentially catastrophic global carbon cycle imbalances with ‘the potential to significantly alter the earth system within a relatively short time frame’ (Tng *et al*., 2012a). Chapman & Harrington (1997) stated that ‘to maximize biological diversity in the wet tropics of north Queensland, it is necessary to maintain the full spectrum of natural habitats. Fire management is therefore required to maintain the wet eucalypt forest and its dependent fauna’. Chapman & Kofron (2010) wrote that ‘unless fire has a central role in managing tropical wet sclerophyll forest, then this forest type and its dependent species will cease to exist’.

Although we acknowledge the detailed spatial analyses required to document changing forest boundaries, the ‘invasion’ argument has numerous issues. It often omits historical context, uses post‐clearing baselines of forest boundaries, labels native plants as ‘invasive,’ emphasizes fire interventions, and selectively focuses on protecting a few tropical TEF vertebrate species (Table 1) at the apparent expense of the greater vertebrate diversity, endemism, and threatened species concentrations in adjacent rainforests (Nix & Switzer, 1991; Williams *et al*., 2010; WTMA, 2016). The Wet Tropics contain 45% of all Australian vertebrate species, and critical conservation areas for rainforest animals are concentrated on the western edge (WTMA, 2016), where the effects of human modification of the rainforest–TEF interface are the highest. Despite the urgent warnings, little evidence indicates that rainforest edge movements or insufficient burning has caused any biodiversity catastrophe, and there are few monitoring studies (Bradford & Harrington, 1999; Tng, 2019). By contrast, excessive fire, logging, agriculture, clearing, and invasive predators are well‐documented threats to tropical TEF (and rainforest) species, including, ironically, the vertebrates most commonly stated to require increased rainforest suppression through burning for survival (Table 1). The logging and burning of large trees remove vital tree‐hollow habitats, a little‐studied factor that has detrimental effects on several TEF species (Woinarski *et al*., 2016; Tng, 2019). The core idea that active fire promotion is required to maintain TEFs lacks experimental support (Fensham & Fairfax, 2002).

**Table 1:** International Union for Conservation of Nature (IUCN)‐assessed Red List status and top threats for selected vertebrate species found in tall eucalypt forests.

Common name	Scientific name	Red List status, trend	Threats
Yellow‐Bellied Glider	*Petaurus australis*	Near Threatened, decreasing	Logging, agriculture, other clearing, fire
Mahogany Glider	*Petaurus gracilis*	Endangered, decreasing	Agriculture, aquaculture, invasive weeds, altered fire regime
Greater Glider	*Petauroides volans*	Vulnerable, decreasing	Logging, agriculture, increased fire, climate change
Northern Bettong	*Bettongia tropica*	Endangered, decreasing	Feral cats, drought, increased fire, agriculture
Hastings River Mouse	*Pseudomys oralis*	Vulnerable, decreasing	Feral cats, Red Foxes, logging, increased and suppressed fire
Stephen's Banded Snake	*Hoplocephalus stephensii*	Near Threatened, decreasing	Logging, agriculture, other clearing, fire
Eastern Bristlebird	*Dasyornis brachypterus*	Vulnerable, decreasing	Increased fire, climate change

Threats and fire trends listed are those highlighted in the respective IUCN species assessments (IUCN, 2023). Several species noted in the literature are not listed here because they belong to the Least Concern category, including Eastern Yellow Robin, Yellow Thornbill, Buff‐Rumped Thornbill, and Powerful Owl, some of which are threat‐listed at the state level.

Historical decreases in Aboriginal fire management have been invoked as the principal justification for fire suppression in many of the listed papers. However, Aboriginal practices provide questionable equivalence for justifying programmed burning because of their vastly different objectives, seasonal timing, and scale (Hill *et al*., 2000). Australia's tropical rainforests also have high biomass, often exceeding that of the TEFs (Unwin, 1989; Bradford *et al*., 2014; Wardell‐Johnson *et al*., 2017). Suppressing them with fire puts Australia at odds with the world‐wide consensus for conserving biodiverse, carbon‐dense rainforests (e.g. Muthee *et al*., 2022; Mo *et al*., 2023) to prevent extinction and forest conversion from carbon sinks to sources (Baccini *et al*., 2017), a process accelerated by excessive fire (Datta & Krishnamoorti, 2022).

In contrast to the ‘invasive’ narrative, other literature characterizes Australian rainforests more broadly to include the TEFs not as a distinct, adversarial forest type but as an intermittent disturbance zone dominated by rainforest pioneer plants, which in Australia overwhelmingly consist of the charismatic, frequently enormous eucalypts (van Steenis, 1958; Smith & Guyer, 1983; Adam, 1992; Tng *et al*., 2012b, 2014; Lindenmayer *et al*., 2024). Long‐lived, opportunistic rainforest pioneer trees world‐wide, including Australian eucalypts, were well defined by van Steenis (1958) as ecological ‘nomads,’ which take advantage of bare soils exposed by processes including volcanic flows, erosion, fires, landslides, and anthropogenic clearing. Leaf traits analyzed across TEF–rainforest transitions showed no significant differences (Tng *et al*., 2013), further reducing the distinctiveness of TEFs from rainforests regarding functional ecology. In this context, tropical TEFs have been proposed as rainforest fringe habitats dominated by eucalypt nomads that evolved pioneer‐like traits under various nonfire disturbance regimes (Smith & Guyer, 1983).

Advancing the debate about tropical TEFs requires historical context from the fossil record, which has often been invoked as a missing or data‐deficient element (e.g. Hopkins, 1981; Unwin, 1989; Adam, 1992; Wardell‐Johnson *et al*., 1997) but now has much to offer from recent discoveries. We now turn to fossil data to address fundamental questions. Were eucalypts associated with rainforests through geologic time? If so, is human intervention necessary today? Have most eucalypts always been fire‐dependent, or were they exapted (preadapted) to fire in earlier settings? What can we learn from the fossil record to improve forest conservation? We review the fossil history of eucalypt‐rainforest interactions and show that Australian tropical rainforests and TEFs represent a modern iteration of a single interacting system that can be traced back to at least 50 million years ago (Ma) in Gondwana, with no evidence of significant fire mediation until geologically recent times. Based on the fossil data, we reject the ‘invasive’ narrative and conclude by highlighting the need for improved modern conservation and management of the demonstrably ancient, evolutionarily significant rainforest–TEF interface.

## Eucalypts in rainforests through time

### Eocene Patagonia

**Fig. 2:**
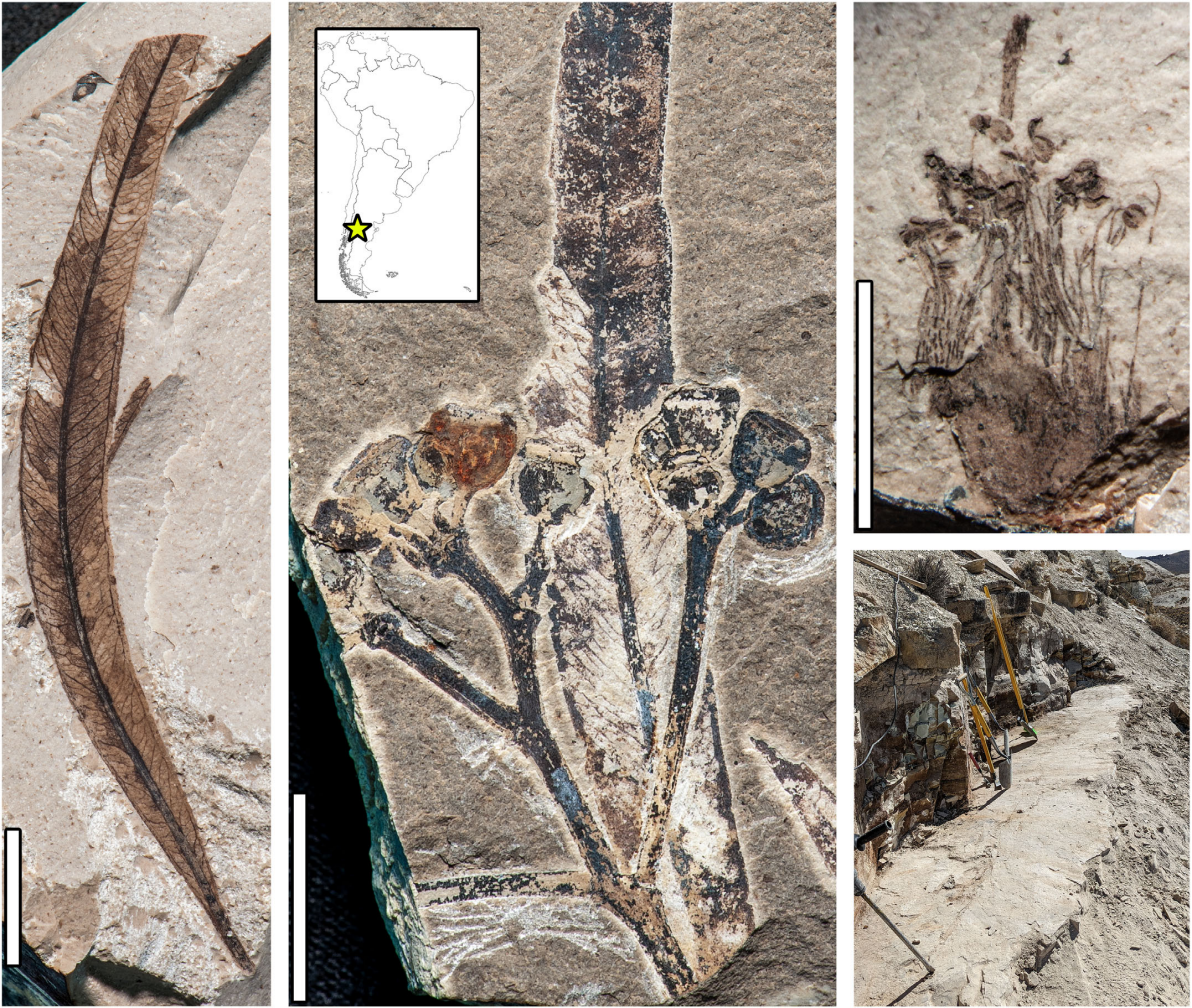
Early Eocene *Eucalyptus* fossils from Laguna del Hunco, Chubut, Argentina (star on the map inset; see Gandolfo *et al*., 2011; Hermsen *et al*., 2012; Zamaloa *et al*., 2020). From left, *E. frenguelliana* holotype, MPEF‐Pb 2329 (quarry LH4), bar, 1 cm; *E. caldericola* holotype (infructescence), MPEF‐Pb 5021, and *E. frenguelliana* leaf (quarry LH25), bar, 1 cm; holotype of *E. xoshemium* (flower) with *in situ Myrtaceidites eucalyptoides* pollen, MPEF‐Pb 5022 (quarry LH2), bar, 5 mm. Multiple names are used to denote the dispersed organs, but there is no evidence of more than one biological species of *Eucalyptus* at the site. Bottom right, a fresh excavation at quarry LH13, a prolific location for *Eucalyptus* fossils, showing the challenges of accessing thin productive strata under several meters of indurated overburden. MPEF‐Pb, Paleobotanical Collections of Museo Paleontológico Egidio Feruglio, Trelew, Chubut, Argentina.

**Fig. 3:**
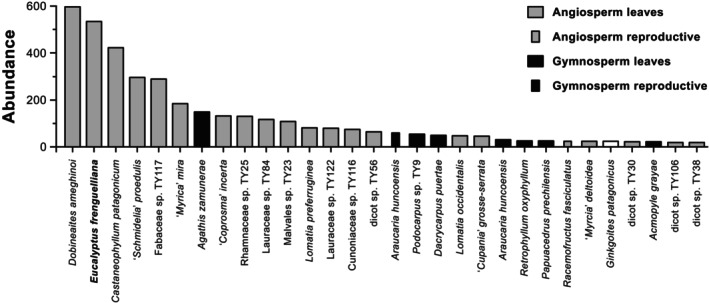
Top 30 most abundant plant organs (of > 200 known) at the early Eocene Laguna del Hunco site in Chubut, Argentina, based on raw counts of 4303 total fossils from all quarries combined, nomenclature updated from Wilf *et al*. (2005). Abundant families include Anacardiaceae (*Dobineaites*), Myrtaceae (*Eucalyptus* and ‘*Myrcia*’), Fagaceae (*Castaneophyllum*), Fabaceae, Araucariaceae (*Agathis* and *Araucaria*), Rhamnaceae, Lauraceae, Proteaceae, Cunoniaceae (sp. TY116 and *Racemofructus*, considered related to *Weinmannia* s.l.), Podocarpaceae (*Podocarpus*, *Dacrycarpus*, *Retrophyllum*, *Acmopyle*), and Cupressaceae (*Papuacedrus*).

Until recently, the eucalypt fossil record was sparse and confined to Australia and New Zealand, limiting direct insights into the group's early history and biogeography (e.g. Hill *et al*., 2016). This situation changed with the recent discovery of the oldest and best‐preserved *Eucalyptus* fossils at the early Eocene (52 Ma) Laguna del Hunco fossil lake site in Chubut, Patagonian Argentina (Fig. 2). Fossil *Eucalyptus frenguelliana* leaves are preserved there in great abundance (Fig. 3), along with extraordinary additional evidence for the genus from complete infructescences, flower buds, and a flower with *in situ* pollen (Fig. 2; Gandolfo *et al*., 2011; Hermsen *et al*., 2012; Zamaloa *et al*., 2020). The combined evidence is sufficient to place the fossils in the *Eucalyptus* crown group, and they preserve diagnostic features of the large subgenus *Symphyomyrtus*.

*Eucalyptus* leaves, preserving characteristic oil bodies and occasionally still attached to twigs, were the second most abundant (12% of specimens) of the > 200 plant macrofossil species collected from several thousand fossils at the site (Fig. 3; Wilf *et al*., 2005). These abundance data were obtained from quantitative field counts, and modern leaf litter studies support the idea that the ranked abundance of fossil leaves reflects the relative biomass of their respective source trees (Burnham *et al*., 1992). Therefore, *E. frenguelliana* trees most likely were abundant and reached large sizes. Their ecological importance is also evident from the diverse insect‐feeding damage observed in the leaf fossils, of which every fossil damage type has been found in living Australian wet‐forest eucalypt species, suggesting remarkable conservatism and host tracking (Giraldo *et al*., 2024). Laguna del Hunco is the only South American location that unambiguously preserves fossils of *Eucalyptus* (reviewed by Hermsen *et al*., 2012) and many other genera. The site's floristic distinctiveness presumably reflects both its outstanding fossil preservation and its status as the only well‐sampled paleobotanical site in Patagonia (and South America) from the early Eocene, illuminating the long (*c*. 16 Myr) regional gap between the early Paleocene Salamanca/Las Flores and middle Eocene Río Pichileufú floras (e.g. Wilf *et al*., 2013).

The 170 m thick section of fossil lake beds exposed at Laguna del Hunco belongs to the Tufolitas Laguna del Hunco of the Huitrera Formation and consists mainly of tuffaceous clastic sediments (Aragón & Mazzoni, 1997; Wilf *et al*., 2003; Gosses *et al*., 2021; Hajek *et al*., 2024). The Tufolitas filled a caldera lake of considerable size (*c*. 30 km N‐S) that existed for *c*. 200 Kyr. Plant material was deposited principally through slope failures of the surrounding, steep caldera walls due to earthquakes, overwet soils, and overgrown vegetation; subsequent rapid transport to the lake bottom allowed excellent preservation of diversity and morphological details (Hajek *et al*., 2024). The age of the section is tightly constrained to the 52.2–52.0 Ma window by multiple ^40^Ar‐^39^Ar and U–Pb analyses on primary tuffs from several levels interbedded with the fossiliferous strata and associated igneous rocks (Wilf *et al*., 2003; Gosses *et al*., 2021; Hajek *et al*., 2024). This precise determination dates the fossils to the early Eocene climatic optimum and the terminal phase of Gondwana, the last time a moist forest corridor existed from Patagonia across frost‐free Antarctica to Australia (Wilf *et al*., 2013).

An extraordinary diversity of other well‐preserved plant and animal life occurs in the same beds as the *Eucalyptus* fossils, providing many additional connections to living forests. Notably, many Laguna del Hunco fossil plant taxa now inhabit Australian rainforests and interact directly with *Eucalyptus*, including the fern *Todea*; the conifers *Agathis*, *Araucaria*, and *Podocarpus*; angiosperms such as *Ceratopetalum*, *Gymnostoma*, *Orites*, *Macaranga*, *Akania*, and *Ripogonum*; and others closely related to *Daphnandra*, *Wilkiea*, *Zygogynum*, and *Ackama* (for summaries and primary references, see Wilf *et al*., 2005, 2019, 2023; Barreda *et al*., 2020; Matel *et al*., 2022). Additional Laguna del Hunco genera are also well known as fossils from Australia but survive elsewhere, including the abundant (Fig. 3) water‐demanding conifers *Papuacedrus*, *Acmopyle*, *Dacrycarpus*, and *Retrophyllum*, whose extinction in Australia is attributed to increasing water stress (Brodribb & Hill, 2004). These taxa comprise some of the numerous examples of how Australia served as the ‘life raft’ enabling the survival of multiple Gondwanan paleo‐Antarctic rainforest lineages that persisted there or eventually dispersed to island Australasia, Malesia and beyond (Kooyman *et al*., 2014; Wilf *et al*., 2019). The climate was frost‐free and everwet (perhumid), inferred from the drought intolerance of many living relatives of the plants and confirmed by the extraordinary preservation of highly drought‐sensitive accessory transfusion tissue in *Acmopyle* leaves (Andruchow‐Colombo *et al*., 2023) and vessel‐less Winteraceae wood (Brea *et al*., 2021).

Reconstructing the landscape ecology of the Patagonian fossil eucalypts is challenging because the remains were transported into a large lake basin with no information regarding their original locations. However, they co‐occurred on the same bedding planes as all the taxa listed, which means that they were deposited during the same transport events and grew close to or intermingled with traditional rainforest elements. The environment was dynamic, and the steep volcanic habitat provided significant disturbances (Hajek *et al*., 2024); we suggested earlier (in Gandolfo *et al*., 2011) that the ancient *Eucalyptus* trees were opportunists that readily colonized areas with freshly exposed mineral soils. Today, several non‐Australian rainforest eucalypts specialize in nonfire disturbances, including volcanic clearance, fluvial erosion, landslides, and clear‐cuts, such as the Rainbow Gum (*E. deglupta*) in New Guinea (Carr, 1972; Paijmans, 1976; Johns, 1986). Even in Australia, eucalypts recruit in gaps, and cyclones frequently damage large areas of tropical rainforest, a process thought to facilitate eucalypt recruitment at the margins, although direct documentation is scarce (Unwin *et al*., 1988; Tng *et al*., 2012a, 2012b). As in New Guinea's volcanic province and similar modern environments (Ganteaume & Syphard, 2018), fires at Laguna del Hunco would have been rare and limited to lightning strikes and volcanic ignition during active eruptions, as corroborated by the lack of definite charcoal in the site sediments (Barreda *et al*., 2020; V. Barreda, pers. comm. 2024). The caldera slopes probably featured a complex mosaic of regenerating forest patches from mixed disturbance cohorts, as seen today on New Guinea landslide surfaces (Johns, 1986).

From the high abundances of both *Eucalyptus* and standard rainforest elements and their persistence throughout the 200 Kyr window of fossil deposition (Wilf *et al*., 2005; Fig. 3), we see that neither rainforests nor eucalypts extinguished the other. Instead, natural expansion, contraction, disturbance, and regeneration maintained a single interacting system. The caldera lake margin forests included diverse life‐history strategies embodied by *Eucalyptus* and a few probable others (*Macaranga*, *Physalis*, and Gleicheniaceae) as disturbance specialists, whereas most taxa represented closed forests. However, the closed forest category is more heterogeneous than is often assumed; disturbance opportunism in Australia, New Guinea, and elsewhere is well known in several conifer and angiosperm genera represented by fossils at Laguna del Hunco (e.g. Johns, 1986, 1990; Enright & Hill, 1995). Pole (2019) recently suggested the alternate hypothesis that Laguna del Hunco eucalypts inhabited closed forests. This explanation is intriguing but less likely because of the direct sedimentological evidence for multiple disturbance types, including frequent slope failures associated directly with fossil deposition events (Hajek *et al*., 2024), which would have limited the area of closed forests and provided abundant fresh mineral soil exposures for recruitment. The vegetative and reproductive traits in the *Eucalyptus* fossils are virtually indistinguishable from their living counterparts (including foliar oil bodies, operculate corollas, and valved, capsular fruits; Hermsen *et al*., 2012), implying conserved life‐history strategies.

### Australia and New Zealand

Despite a large number of paleobotanical localities (e.g. Hill, 1994; Kooyman *et al*., 2014), the eucalypt macrofossil record from Australia and New Zealand remains limited, especially for *Eucalyptus* s.s. However, *Eucalyptus* s.l. leaves and a few fruit capsules have been recorded since the early Eocene in Australia, and several incompletely studied reproductive fossils hold promise for detailed investigations using new techniques (Rozefelds, 1996; Hermsen *et al*., 2012; Hill *et al*., 2016; Pole, 2019; Thornhill *et al*., 2019; Rozefelds *et al*., 2024). The oldest well‐accepted occurrence of *Eucalyptus* s.s. consists of leaves from Berwick Quarry (Victoria, late Oligocene/early Miocene; Pole *et al*., 1993; Hill *et al*., 2016). Neogene and younger *Eucalyptus* reproductive fossils are known from Chalk Mountain, Miocene of New South Wales (Holmes & Anderson, 2019), Bacchus Marsh, late Miocene of Victoria (Ladiges, 1997), Stony Creek, early Pleistocene of Victoria (e.g. Sniderman *et al*., 2013), and various Cenozoic fluvial and basaltic silcrete deposits (Rozefelds *et al*., 2024).

There are several informative patterns in the scarce record. First, even the best‐studied Paleogene sites lack definite evidence of eucalypts, such as Anglesea, Golden Grove, and Cethana (e.g. Hill, 1994). Second, unlike Laguna del Hunco, *Eucalyptus* macrofossils are not reported to be abundant at any time in Australia, except for a leaf mat from Berwick Quarry (Pole *et al*., 1993). The excellent preservation of abundant *Eucalyptus* at Laguna del Hunco in Argentina (Figs 2, 3) indicates no intrinsic preservation bias against this genus, in contrast to some taphonomic studies (e.g. Hill & Gibson, 1986). Thus, we consider the overall rarity of Paleogene *Eucalyptus* macrofossils in Australia to reflect low original abundance, at least locally, although palynological evidence for eucalypts is more common (Thornhill *et al*., 2019).

Third, eucalypt macrofossils from Australia and New Zealand almost always co‐occur with typical rainforest elements. For example, Berwick Quarry preserves *Dacrycarpus*, *Gymnostoma*, and *Nothofagus*, leading to the interpretation of a mixture of rainforests and disturbance‐mediated open forests in a volcanic landscape with no evidence of fire (Pole *et al*., 1993; Hill *et al*., 2016), a setting comparable to Laguna del Hunco. Several other Australian fossil sites with eucalypts have associated volcanic strata (Rozefelds *et al*., 2024), and volcanism was widespread in eastern Australia from the Eocene to the Miocene, continuing into the Pleistocene in tropical Queensland (Jones *et al*., 2017). Like Laguna del Hunco, volcanic eruptions, extrusive flows, ash falls, and landslides were presumably important factors in forest disturbance, whereas fire ignition was mostly limited to lightning strikes (Enright & Thomas, 2008). Rainforest elements are also present at Neogene‐Pleistocene sites that preserve *Eucalyptus* capsules, including Chalk Mountain (*Agathis*, *Ceratopetalum*, *Dendrocnide*; Holmes & Anderson, 2019) and Bacchus Marsh (*Nothofagus*, *Araucaria*, *Dacrycarpus*; Greenwood, 1994). Nearly all these associated genera (except *Dendrocnide*) are reported from Laguna del Hunco (Barreda *et al*., 2020). The combined evidence from the austral records suggests that *Eucalyptus* has spent much of its history as a rainforest disturbance specialist in largely nonfire disturbance regimes.

Fourth, it is striking that *Eucalyptus* is, thus far, not known from nearly all pre‐Quaternary assemblages with well‐characterized sclerophyll or open forest signatures, including several Paleocene and younger sites that preserve well‐vetted fossils of the classical sclerophyll element *Banksia* (e.g. Carpenter *et al*., 2014, 2016; McCurry *et al*., 2022). Some localities preserve direct evidence of fire disturbance in charcoal records, such as the Eocene–Miocene Latrobe sequence in Victoria (Korasidis *et al*., 2019), or xeromorphic adaptations, as seen in fossil *Banksia* leaf anatomy (Carpenter *et al*., 2014).

*Eucalyptus* reached high abundances in Australian pollen assemblages by the late Miocene to early Pliocene (e.g. Nullarbor Plain, southwestern Australia; Sniderman *et al*., 2016), approximately coeval with increased fire, as indicated by charcoal counts (Kershaw *et al*., 1994). *Eucalyptus* involvement in fire‐prone sclerophyll assemblages is well‐captured in a geologically young and well‐preserved early Pleistocene deposit at Stony Creek, Victoria. (Sniderman & Haberle, 2012; Sniderman *et al*., 2013). The Stony Creek macrofossil and pollen floras preserve a nonanalog, diverse, humid sclerophyll forest type, wherein charcoal frequency is correlated positively with periods of *Eucalyptus* pollen dominance and negatively with rainforest intervals. Thus, Stony Creek is the oldest well‐preserved example of interacting fire, rainforests, and sclerophyll vegetation with confirmed *Eucalyptus* macrofossils, demonstrating natural fire response and survival of Australian rainforest and sclerophyll vegetation together in a prehuman, *c*. 1.5 Ma, mixed‐forest setting.

There are no relevant *Eucalyptus* macrofossils from dryland biomes because of well‐known preservation biases. However, a calibrated molecular study detected robust signals for species diversification across five sections of subgenus *Symphyomyrtus*, beginning only *c*. 3 Ma (Thornhill *et al*., 2019). This result aligned well with isotopic evidence from leaf waxes in marine cores off northwest Australia for an increase in Australian C_4_ plant abundance, considered related to increased rainfall seasonality and fire, at *c*. 3.5 Ma, later than most other continents (Andrae *et al*., 2018). Similarly, evidence from the sedimentary geochemistry of river muds from Western Australia showed significant fluctuations in moisture since 5.3 Ma and evidence for increasing aridity and rainfall seasonality since *c*. 3.8 Ma (Stuut *et al*., 2019).

Detailed information about the eucalypt‐rainforest‐fire system since the late Pleistocene primarily comes from pollen, charcoal, and other proxy data from volcanic maar lakes in northern Queensland and the spectacular discovery of subfossil *Eucalyptus* charcoal in the Daintree rainforest (Hopkins *et al*., 1993, 1996). These datasets show significant contractions, fragmentations, and re‐expansions of rainforest and eucalypt forests through time (e.g. Kershaw, 1994; Dimitriadis & Cranston, 2001; Haberle, 2005; Kershaw *et al*., 2007; Haberle *et al*., 2010). However, there has been little assessment of volcanic disturbance to the paleovegetation in this area, which includes extensive Quaternary basalt deposits and maar lakes (Dimitriadis & Cranston, 2001). The potential effects of Aboriginal populations on forest dynamics have been extensively discussed, especially regarding the promotion of fire (Cremer, 1960; Kershaw, 1994; Hopkins *et al*., 1996; Kershaw *et al*., 2007). However, there is scarce evidence that Aboriginal fire management had transformative impacts in wetter regions of the continent (Enright & Thomas, 2008; Roberts *et al*., 2021; Lindenmayer *et al*., 2024). Most large Aboriginal fires observed by early settlers were set for resistance purposes, not to clear land (Hill *et al*., 2000). In the Wet Tropics, some Aboriginal fire practices that conserve localized resources in closed and open forests remain (Hill *et al*., 2000; Roberts *et al*., 2021).

The profound impacts on Australian forests since European colonial activities include those of agriculture, grazing, logging, introduced species, mining and programmed burning (e.g. Hill *et al*., 2000; Bradshaw, 2012; Fig. 1). Queensland has lost *c*. 50% of its tropical forests and 74% of its lowland and tableland rainforests since European arrival, mainly for agriculture, and it currently loses more forest annually than any other Australian state (Bradshaw, 2012; Neldner *et al*., 2023). Queensland's rainforests were heavily cleared because they were thought to hold the most fertile soils, and their foliage was considered a food source for beetles that fed on sugarcane crops; some supposedly harmful rainforest ‘expansion’ represents natural recovery from massive sugarcane clearing and mining (Hill *et al*., 2000, 2001). Today, many examples of purported rainforest ‘encroachment’ that are linked to current fire‐management policies (Harrington & Sanderson, 1994; Russell‐Smith *et al*., 2004; Tng *et al*., 2012a; Stanton *et al*., 2014; Krishnan *et al*., 2019) appear to be measured against postclearing baselines. It is more likely that the ‘encroachment’ represents small recoveries from earlier, large‐scale deforestation (Hill *et al*., 2000) that removed twice the coverage of rainforests vs TEFs (Fig. 1). Cattle and horse grazing, often combined with fire management, are significant factors that increase the loss rate of TEFs (by nine times in one study) through the removal of grass at the rainforest margin, expediting recruitment by rainforest plants (Unwin, 1989; Hill *et al*., 2001; Campbell & Clarke, 2006).

## Discussion

Large, emergent eucalypts in or around rainforest assemblages can be explained either by reclassifying these areas as nonrainforest due to eucalypt presence or by recognizing that eucalypts reflect interactions between shade‐tolerant, long‐lived rainforest trees, and opportunistic, long‐lived, shade‐intolerant pioneers that regenerate on disturbed mineral soils. The strategy of long‐lived opportunists (nomads; see the Introduction section), with numerous analogs globally, is to arrive early under high‐light conditions with mineral soil exposure, with initially low levels of competition, and to leave late, persisting in the stand as tall emergent trees (Fig. 1).

Postdisturbance rainforest pioneers, other than eucalypts, are often described as having a mix of traits associated with short lifespans, such as low wood density, low stature, small seeds, shade intolerance, and low leaf mass per area; however, other early secondary and opportunist nomad‐type species (e.g. some *Cecropia* species in the Neotropics; *Dendrocnide* and *Flindersia* in Australia) can germinate quickly after disturbance, grow rapidly, and persist as large trees. Eucalypts have traits that allow them to become some of the largest plants world‐wide, reflecting similar, opportunistic ecological strategies. Forest dynamics can favor plant traits and strategies differentially, including the effects of long‐time‐interval disturbances and expansion‐contraction driven by climatic oscillations. Under some circumstances, these processes lead to the intermixing of plant ecological strategy types, reflecting short and long regeneration intervals and long‐timescale biogeographic patterns.

The fossil evidence shows that *Eucalyptus* has been an integrated disturbance specialist in rainforest assemblages for most of its known history, suggesting that Australia's TEFs are a modern, fire‐mediated iteration of ancient rainforest disturbance zones dominated by eucalypts that extended far beyond Australia. Until perhaps the past 5 Myr, the disturbances largely came from nonfire causes, and eucalypts apparently were absent from sclerophyll vegetation. Disturbances that create opportunities by allowing natural contraction and expansion of rainforests have resulted from volcanic activity and dormancy, as seen in ancient Patagonia, modern New Guinea, and Eocene–Miocene Australia, as well as landslides, fluvial processes, and climate change. The emergence of fire disturbance in the late Cenozoic of Australia was associated with the natural contraction and expansion of rainforests and eucalypt forests, but not with the extirpation of either (Sniderman & Haberle, 2012).

The close affinities of eucalypts with rainforest Myrtaceae (e.g. *Eucalyptopsis*, *Stockwellia*; Ladiges *et al*., 2003; Thornhill *et al*., 2019) have long suggested that they originated in humid habitats with limited fire. However, a molecular phylogenetic study found that epicormic resprouting, a signature fire adaptation in living eucalypts, maps deeply onto the phylogeny of living Australian *Eucalyptus* species, supporting the view that fire ecology has been prevalent in the crown lineage since the Cretaceous (Crisp *et al*., 2011). This conclusion rested on the assumptions that fire adaptation in deep time can be inferred, without fossils, from mapping one trait onto molecular phylogenies and that fire has always been the singular stimulus for the trait. Nevertheless, epicormic resprouting also expedites regenerative responses in eucalypts and other plants to nonfire disturbances (Burrows, 2013; Kenefick *et al*., 2024), all of which are applicable to early eucalypts, including mechanical injuries (landslides, wind, and herbivores), droughts, and lava flows.

Overall, the evidence indicates that early wet‐forest eucalypts were nomads adapted to nonfire disturbance, enabling them much later in their history to take remarkable advantage of novel, expanding fire regimes by exaptation (van Steenis, 1958; Smith & Guyer, 1983). The Eocene Laguna del Hunco fossils occur in a highly disturbed paleoenvironment with frequent mineral soil exposure (Hajek *et al*., 2024) and without evidence of fire. They preserve foliar oil bodies, which are implicated today in herbivore deterrence and increased flammability, and thick‐walled capsular fruits that confer seed protection, whose living analogs allow postfire seed release (e.g. Hermsen *et al*., 2012; Hernández *et al*., 2022).

We view the tropical TEF–rainforest interface as an ancient evolutionary system that provides ample but underappreciated fulfillment of World Heritage criteria *viii* and *ix* for conserving evolutionary processes and stages in the evolution of life (https://whc.unesco.org/en/criteria). These valuable forests should be restored and conserved (Tng *et al*., 2014; Lindenmayer *et al*., 2024) to support species fitness and resilience via evolution through natural selection at the rainforest edge. We find no historical or experimental basis for the established but long‐disputed (Gilbert, 1959) idea that fire intervention is required to maintain rainforest–TEF boundaries (see Fensham & Fairfax, 2002). This contention is directly refuted by charcoal and pollen data from Pleistocene Australia, which show that well before human influence, rainforests and eucalypt forests intermingled and regenerated over time in response to fire regimes (Sniderman & Haberle, 2012). We see no justification for the widespread burning of the margins of Australia's remaining rainforests (Fig. 1) to make even more space for *Eucalyptus*, a single genus that already dominates 77% of native Australian forests (ABARES, 2018), than it would occupy as part of the natural expanding and contracting rainforest systems that existed for tens of millions of years before humans.

We partly agree with Chapman & Harrington (1997) that ‘to maximize biological diversity in the wet tropics of north Queensland, it is necessary to maintain the full spectrum of natural habitats,’ but this should involve restoring and not intensively burning the specific habitat of dynamic rainforest margins. Oxymoronic terminology that labels native tropical rainforests as ‘invasive’ and ‘encroaching’ is deeply ingrained in the literature and forestry practice. This viewpoint reflects a short‐term perspective that overlooks deep time and human history, natural forest dynamics, and the disproportionate historical and continuing losses of Australian rainforests compared to TEFs (Fig. 1; Ash, 1988; Fensham & Fairfax, 2002; Bradshaw, 2012; Hansen *et al*., 2013; Tng, 2019; Bauman *et al*., 2022). Little evidence suggests that rainforest expansions have reduced biodiversity or population sizes except at limited spatial scales (Baker *et al*., 2020). By contrast, clearing, logging, increased fire, exclusion fencing, barbed wire, invasive weeds, and carnivores are well‐documented sustained threats to tropical wildlife over large areas (Table 1).

## Outlook

We recommend revised management policies to re‐establish the natural dynamics and demographics of the interface zone, prioritizing both natural rainforest recovery and the integrity of TEFs, with reduced logging to allow mixed forests with charismatic and ecologically significant giant eucalypts to re‐emerge. Precise measures require discussion, development, monitoring, economic planning, and interdisciplinary investigations, and we avoid being prescriptive. However, additional actions could prioritize minimized grazing at the interface and emphasize strategic, small‐scale fire management, in close consultation with the First Nations, to protect forest dynamics, essential wildlife habitats, and traditional knowledge and ways of life. Rainforests should not be used as firebreaks; burning should instead be directed away from the rainforest. Promising scientific avenues include intensified gathering of historical data and modeling to improve the precision and widespread use of presettlement, not postclearing baselines (Fig. 1); increased monitoring studies for endangered species at the interface; assessments of multiple disturbance types on fire characteristics, stand demographics, and biotic interactions; and precise quantification of changes in three‐dimensional burned and unburned forest structures over time using remote sensing and terrestrial lidar scanning (Forbes *et al*., 2022; Lindenmayer *et al*., 2022). The rewards will be considerable, including survival and significantly increased health of ancient rainforests and TEFs, thereby increasing the value of Australian contributions to biodiversity and World Heritage.

## Competing interests

None declared.

## Author contributions

PW and RMK developed the ideas presented here and drafted the manuscript collaboratively.

## Data Availability

All data discussed here are available in the cited literature.
